# Evaluation of Mycotoxin Residues on Ready-to-Eat Food by Chromatographic Methods Coupled to Mass Spectrometry in Tandem

**DOI:** 10.3390/toxins10060243

**Published:** 2018-06-15

**Authors:** Dionisia Carballo, Guillermina Font, Emilia Ferrer, Houda Berrada

**Affiliations:** 1 Faculty of Agricultural Science, National University of Asunción, San Lorenzo 2160, Paraguay; diocarve@alumni.uv.es; 2 Laboratory of Food Chemistry and Toxicology, Faculty of Pharmacy, University of Valencia, Avenue Vicent Andrés Estellés s/n, 46100 Burjassot, Spain; Guillermina.font@uv.es (G.F.); Emilia.ferrer@uv.es (E.F.)

**Keywords:** mycotoxins, ready-to-eat food, GC-MS/MS, LC-MS/MS, Valencia

## Abstract

Simultaneous determination of twenty-seven mycotoxins in ready-to-eat food samples using “Quick Easy Cheap Rough and Safe” (QuEChERS) extraction and chromatographic methods coupled to mass spectrometry in tandem is described in this study. Mycotoxins included in this survey were aflatoxins (B_1_, B_2_, G_1_, G_2_), enniatins (A, A_1_, B, B_1_), beauvericin (BEA), fumonisins (FB_1_, FB_2_), sterigmatocystin (STG), deoxynivalenol (DON), 3-acetyl-deoxynivalenol (3-ADON), 15-acetyl-deoxynivalenol (15-ADON), nivalenol (NIV), neosolaniol (NEO), diacetoxyscirpenol (DAS), fusarenon-X (FUS-X), zearalenone (ZEA), α-zearalanol (αZAL), β-zearalenone (βZAL), α-zearalenol (αZOL), β-zearalenol (βzol), T2, and HT-2 toxin. The method showed satisfactory extraction results with recoveries ranging from 63 to 119% for the different food matrix samples. Limits of detection (LOD_S_) and quantification (LOQs) were between 0.15–1.5 µg/kg and 0.5–5 µg/kg, respectively. The method was successfully applied to the analysis of 25 ready-to-eat food samples. Results showed presence of deoxynivalenol at 36% of samples (2.61–21.59 µg/kg), enniatin B at 20% of samples (9.83–86.32 µg/kg), HT-2 toxin at 16% of samples (9.06–34.43 µg/kg), and aflatoxin G_2_ at 4% of samples (2.84 µg/kg). Mycotoxins were detected mainly in ready-to-eat food samples prepared with cereals, vegetables, and legumes, even at levels below those often obtained from raw food.

## 1. Introduction

Mycotoxins are a group of toxic compounds produced as secondary metabolites by certain fungi of the genus *Aspergillus*, *Penicillium*, *Fusarium*, *Alternaria*, and *Claviceps* that grow under different climate conditions and have been reported in several food matrices like cereals, peanuts, meat, eggs, milk, and fruits [1,2]. Chronic exposure to some mycotoxins can produce carcinogenic, mutagenic, or teratogenic effects. Aflatoxins (AFs) are indicated as carcinogenic and hepatotoxic and fumonisins (FBs) and ochratoxin A (OTA) are possibly teratogenic, hepatotoxic, and nephrotoxic while patulin (PAT), zearalenone (ZEA), deoxynivalenol (DON), nivalenol (NIV), T-2, and HT-2 toxins are related to toxicological effects mainly on gastrointestinal tract, immune, and endocrine systems [3]. Emerging mycotoxins, such as enniatins ENs and beauvericin, are cytotoxic [4] and their potent cytotoxic activity was demonstrated in several mammalian cell lines [5], turning it in a topic for human health [6]. The European Union through Regulation (EC) 1881/2006 set maximum limits in foodstuffs susceptible to mycotoxins contamination to control their quality and safety [7] (Table 1).

The determination of mycotoxins has been highly reported in different food matrices such as cereal products [8], coffee [9], tomatoes and tomato products [10], fruits, vegetables, and legumes [11]. There are several food processing methods that may modify mycotoxin stability [12]. Visconti et al. [13] and Serrano et al. [14] reported gradual reduction of DON and ENs contents in pasta and AFs and OTA decreased in cooked rice through thermal treatments [15].

However, there is limited information on the presence of mycotoxins in ready-to-eat food. Recently, the EFSA (European Food Safety Authority) Panel on Contaminants in the food Chain (CONTAM) emitted a Scientific Opinion recommending that further studies should be conducted on the fate of mycotoxins during the preparation of grain-based products, specially focused in bread, pasta, and fine bakery wares [16].

Chromatographic methods coupled to mass spectrometry are the techniques often used for the determination of mycotoxins in food matrices [17,18]. One of the main objectives pursued in most laboratories is the application of multiple analyte determination with minimal sample treatment. To achieve increased throughput over traditional mycotoxin extraction methods, novel techniques were employed and by using appropriate mixtures of extraction and disperser solvents in dispersive liquid-liquid microextraction (DLLME), and also by adding inorganic salt into a mixture of water and organic solvent in salting-out liquid-liquid extraction (SALLE).

The “Quick Easy Cheap Rough and Safe” (QuEChERS) extraction, originally developed by Anastassiades et al. [19], based on acetonitrile extraction followed by a salting-out and quick dispersive solid-phase extraction (d-SPE), takes advantage of the electric charge of target compounds to allow their extraction with small amounts of non-chlorinated organic solvents. It is also pointed out as a fast and economical mycotoxin extraction method from various processed cereal-based foods [20].

To the best of our knowledge, scarce data exist in the literature concerning mycotoxin level in ready-to-eat food, and in this sense, the development of a simple and efficient method to assess human mycotoxin exposure via ready-to-eat food analysis is necessary.

In the present investigation, an analytical method based on QuEChERS extraction was used to evaluate the presence of twenty-seven mycotoxins AFB_1_, AFB_2_, AFG_1_, AFG_2_, OTA, FB_1_, FB_2_, ENA, ENA_1_, ENB, ENB_1_, BEA, STG, ZON, α-ZAL, β-ZAL, α-ZOL, β-ZOL, DON, 3-ADON, 15-ADON, DAS, NIV, FUS-X, NEO, T-2, and HT-2 in ready-to-eat food by LC-MS/MS and GC-MS/MS.

## 2. Results

### 2.1. Analytical Method Validation

The parameters considered for validation purposes were instrumental linearity, matrix effect, sensitivity (LOD and LOQ), and accuracy according to the EU Commission Decision 2002/657 EC [21]. The criteria for confirmation of positive findings was to fulfil retention time agreement and peak areas ratio obtained from reference standard quantification (Q) and qualification (q) transitions (Table 2).

All mycotoxins exhibited good linearity over the working range (LOQ and 100× LOQ). The regression coefficients of all calibration curves were (r^2^) higher than 0.996, demonstrating a good linearity.

Satisfactory results in terms of recoveries were found for all mycotoxins studied at three fortification levels (25, 50, 100 µg/kg). The range of recovery values for the three concentrations tested in the five food groups studied was between 63% and 119%.

The LODs and LOQs obtained were from 0.15 to 5 µg/kg for all mycotoxins analyzed (Table 2). As can be observed, ENs reached very good sensitivity by LC-MS/MS and DON by GC-MS/MS, achieving LOQs 0.15–0.5 µg/kg, respectively.

The matrix effect (signal enhancement or suppression) was investigated by calculating the percentage ratio between the slopes of the matrix-matched calibration curve and the curve in solvent.

The matrix effect parameter (ME) behavior in the different studied matrix is shown in Table 2. The majority of the analytes showed a signal enhancement, especially trichothecenes (ZON, 3-ADON and NEO) in fish composite with ME of 158%, 173%, and 175%, respectively. On the other hand, signal suppression was registered for AFG1, AFG2, and FB2 in legumes composite with ME values of 53%, 58%, and 59%, respectively. A similar value was obtained for BEA in meat composite with ME of 58%. The matrix-matched standards were used for effective qualifications taking into account results of suppression and enhancement of the signal.

### 2.2. Sample Analysis

Once validated, the method proposed was applied to evaluate the presence of mycotoxins in 25 ready-to-eat food samples. The results obtained are summarized in Table 3.

**Figure 1:**
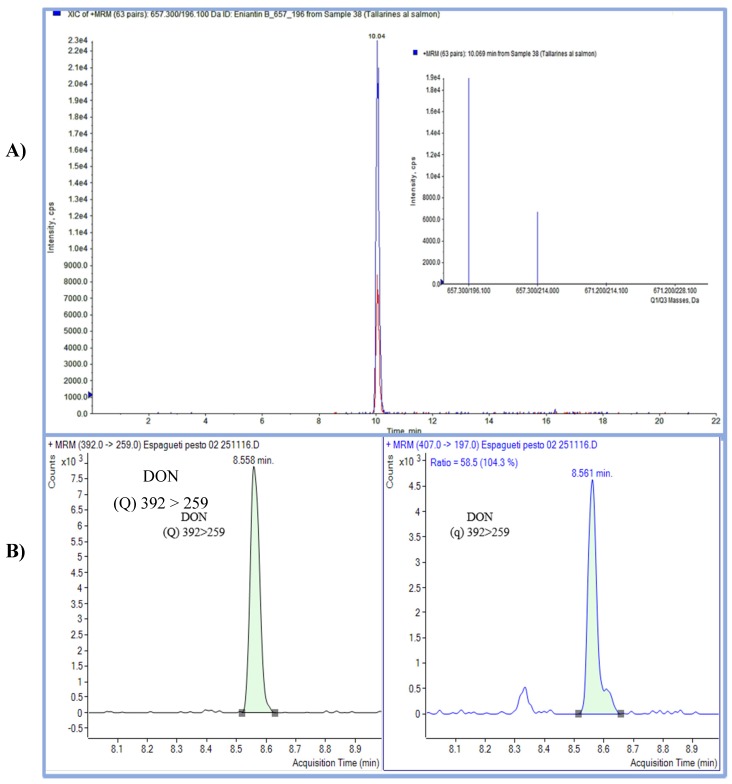
Chromatogram obtained from a sample of ready-to-eat food mainly prepared with cereals and naturally contaminated with (**A**) ENB content 86.32 µg/kg (LC-MS/MS) and (**B**) DON content 21.59 µg/kg at Multiple Reaction Monitoring (MRM) mode by (GC-MS/MS).

Mycotoxins have been detected mainly in food prepared with cereals, vegetables, and legumes. The most prevalent mycotoxin was DON with total incidence of 36% at concentrations of 2.61–21.59 µg/kg being present in pasta, quiche, pizza, garlic soup, and broad beans. ENB was detected in pasta and quiche samples with total incidence of 20% at concentrations of 9.83–86.32 µg/kg. HT-2 toxin was found in pasta and pizza samples with total incidence of 16% at concentrations of 9.06–34.43 µg/kg. Finally, AFG_2_ was evidenced at a concentration of 2.84 µg/kg in one sample of rice. Figure 1 shows the chromatogram from a pasta sample (*n* = 3) naturally contaminated by ENB (86.32 ± 12.2 µg/kg) and DON (21.59 ± 6.2 µg/kg).

### 2.3. Mycotoxin Occurrence in Ready-to-Eat Food Samples

The occurrence of the analyzed mycotoxins in the main food groups studied is presented in Table 4. The samples mainly prepared with cereals showed contamination by DON (88%), ENB (63%), HT-2 (50%), and AFG_2_ (13%). The samples prepared mainly with legumes and vegetables showed DON contamination on 17% and 25%, respectively.

## 3. Discussion

Regarding mycotoxin presence, the contents determined here were comparable with previous research performed in total diet studies (TDS). López et al. [22] reported DON and ENB contents in pasta at concentrations of 8.7 and 35 µg/kg, respectively. Yau et al. [23] also detected DON in a range of 29.95–33.11 µg/kg in pasta, while in vegetables, legumes, and meat samples, the amounts were lower than 5.0 µg/kg. Raad et al. [24] detected DON contents at levels of 62.50 µg/kg in pasta and cereal products, 121.16 µg/kg in pizza, and 31.25 µg/kg in legumes. Sirot et al. [25] detected also DON average of 132 µg/kg in pasta. The results obtained by Beltrán et al. [26] showed occurrence of DON in 100% of pasta and cereal products samples with a maximum value of 203 µg/kg. The concentration of DON in cereals samples never exceed the EU maximum limits of 750 µg/kg for dry pasta [7].

Concerning HT-2, the maximum concentration detected in pasta reached 34.43 µg/kg. Similar amounts were reported by Sirot et al. [25], who quantified HT-2 at levels of 3–10 µg/kg. Leblanc et al. [27] also quantified HT-2 in 238 composite samples with an average level of 270 µg/kg. There are many factors that predispose food to mycotoxin production, such as temperature and storage/processing conditions [28].

In this study, mycotoxins were not found in ready-to-eat meals based on meat and fish. However, Tolosa et al. [29] reported the presence ENs in fish product samples at levels ranging from 1.3 to 103 µg/kg. Sun et al. [30] also suggested that dried seafood could be invaded by mycotoxigenic fungi under improper storage conditions and reported high levels of ZEA in seafood samples (317.3 µg/kg) and OTA (1.9 µg/kg) which were kept for three months at room temperature.

Several studies were carried out on raw food; Cano-Sancho et al. [31] analyzed the presence of mycotoxins in 479 cereal-based food samples and the percentage of DON positive samples in pasta was 73.4% with mean concentration of 226 µg/kg, while HT-2 toxin was present in 10% of samples with mean concentration of 51 µg/kg. Tolosa et al. [32] analyzed 58 samples of different conventional pasta products, the most prevalent mycotoxin was DON (100%) with mean content of 96.93 µg/kg, while HT-2 toxin and ENB were detected in 90% of samples at concentrations from 12.46 to 326.17 µg/kg, respectively. Other mycotoxins were detected with high incidence like NIV, ZEA, ENA_1_, while BEA was present in 10% of analyzed samples.

A recent study conducted by Stanciu et al. [33] did not quantify BEA in 40 pasta samples, while ENB was detected in 11% of the samples at average levels of 10.4 µg/kg and ENB in 9% of the samples at an average level 1.9 µg/kg.

Regarding the presence of emerging mycotoxins in rice, Sifou et al. [34] reported the presence of BEA in 75.5% of samples between 3.8 and 26.3 mg/kg and ENA in 5.7% of analyzed samples with maximum concentration of 448 mg/kg. Makun et al. [35] detected AFs (B_1_, B_2_, G_1_, G_2_) in 100% of rice samples at concentration levels ranging between 28 and 372 µg/kg and OTA in 66.7% of the samples at average level of 141 µg/kg. ZEA was also quantified at 53.4% of the rice samples at an average level 10.6 µg/kg, DON in 28% of the samples at an average level of 18.9 µg/kg, and FB_1_ and FB_2_ were found in 14.3% and 4.8% at concentrations of 0.2 and 6 µg/kg, respectively. Sun et al. [36] reported contents of AFB_1_ in rice and the contents were less than 5 µg/kg, while 7% of samples were detected as exceeding the national maximum residue limit of 10 µg/kg.

On the other hand, some authors have investigated the reduction of mycotoxins during food processing (Table 5); Visconti et al. [13] reported the reduction of DON (until 80%) in pasta by cooking. Cano-Sancho et al. [37] analyzed the transfer of DON from pasta to boiling water, reaching levels of reduction of 75%. Similar values have been reported by Rodriguez-Carrasco et al. [38] who obtained reduction of DON in cooked pasta from 13% to 58%. Nijs et al. [39] also observed a reduction of DON (40%) and ENNs (17–19%) in pasta samples after cooking. Comparable results on ENB reduction levels after the cooking of pasta were reported in another study conducted by Serrano et al. [40].

Castells et al. [41] investigated AFs reduction in rice samples by extrusion-cooking and reported reduction of aflatoxin contents, which ranged from 51% to 95%. Furthermore, Park et al. [43] also reported AFB_1_ loss (78–88%) after pressure cooking. Two different cooking methods were used to evaluate AFs reduction and the authors concluded that steaming of rice samples resulted in the highest aflatoxin reduction (24.8%) [42]. Neither of the methods described reached 100% aflatoxin reduction which highlighted the stability of mycotoxins and their resistance to the different applied processes.

Nevertheless, data obtained show that mycotoxin levels found on ready-to-eat samples are below those often reported for raw food and support the idea that ready-to-eat meal analysis is a suitable alternative for accurate mycotoxin exposure assessment.

## 4. Conclusions

Chromatographic methods coupled to mass spectrometry in tandem were used for evaluation of twenty-seven mycotoxins in ready-to-eat food samples achieving very low limits of quantification. DON was quantified in 36%, ENB in 20%, HT-2 in 16%, and AFG_2_ in 4% of samples, respectively. No mycotoxins were detected in meat and fish dishes. The occurrence of mycotoxins in ready-to-eat food samples was lower than reported on unprocessed cereals, vegetables, and legumes. Exposure assessment from ready-to-eat meals show lowers levels than those obtained from raw food. The evaluation of mycotoxins in ready-to-eat meals offers a reliable tool for risk assessment, since the culinary processes are taken into account.

## 5. Materials and Methods

### 5.1. Chemicals and Reagents

Solvents (acetonitrile, hexane, and methanol) were supplied by Merck (Darmstadt, Germany). Deionized water (<18, MΩcm resistivity) was obtained in the laboratory using a Milli-QSP^®^ Reagent Water System (Millipore, Beadford, MA, USA).

Ammonium formate (99%), formic acid (≥98%), anhydrous magnesium sulphate, and sodium chloride were supplied by Sigma Aldrich (Madrid, Spain). Syringe nylon filters (13 mm diameter 0.22 µm pore size) were obtained from Analysis Vinicos S.L. (Tomelloso, Spain). The derivatization reagent composed of BSA (*N*,*O*-bis(trimethylsily) + TMCS (trimthylcholorosilane) + TMSI (*N*-trimethylsilyimidazole) (3:2:3) was obtained from Supelco (Bellefonte, PA, USA). Sodium dihydrogen phosphate and disodium phosphate, used to prepare phosphate buffer, were acquired from Panreac Quimica S.L.U. (Barcelona, Spain).

### 5.2. Standards and Solutions

The standards of AFB_1_, AFB_2_, AFG_1_, AFG_2_, OTA, FB_1_, FB_2_, ENA, ENA_1_, ENB, ENB_1_, BEA, STG, ZON, α-ZAL, β-ZAL, α-ZOL, β-ZOL, DON, 3-ADON, 15-ADON, DAS, NIV, FUS-X, NEO, T-2, and HT-2 toxins were purchased from Sigma Aldrich. Individual stocks of all analytes were prepared to obtain 20 mg/L in methanol and multianalyte working solutions of 2 mg/L were also used by diluting the individual stock solutions. The multianalyte working standard solution was used for standard calibration curves, matrix-matched calibration curves, and recovery assays. All standards were stored in darkness and kept at −20 °C.

### 5.3. Procedures

#### 5.3.1. Samples

The collection of sample criteria was based on the consumption patterns of the Mediterranean diet, which is characterized by a greater consumption of cereals, vegetables, legumes and fish and less significate amount of meat products CIEAM/FAO [44].

A total of 25 ready-to-eat food samples mainly prepared with cereals (*n* = 8), fish (*n* = 6), legumes (*n* = 5), vegetables (*n* = 4), and meat (*n* = 2) were collected from Valencia University restaurant. For the validation step, 50 g of each black beans, chickpeas, and lentils were cooked and triturated and 2 g of this legume composite were used for QuEChERS extraction. The same pattern was used for all the studied composites. 2 g of cereals composite was taken from 50 g of each bread, pasta, and rice mixture while vegetables composite resulted from 50 g of each of red pepper, green pepper, onions, potatoes, zucchini, tomato, and eggplant. The meat composite was prepared blending 50 g of each of beef, pork, chicken, and finally fish composite resulted from mixing 50 g of each of salmon, perch, and tuna.

Only the edible parts of each food were considered. Seeds and skins were removed from fruit. The inedible part of meat and fish such as bones, thorns, and skin were also removed according to the Commission Regulation EC/401/2006 [45], before analysis performance. All samples were milled with a knife mill (Oster Classic grinder, Valencia, Spain) and the obtained mixture was stored at −18 °C until analysis.

#### 5.3.2. Extraction Procedure

All samples were triturated and homogenized before their analysis. Briefly, 2 g of homogeneous sample were weighed and placed into 50 mL PTFE centrifugal tubes, and then 10 mL of water containing 2% of formic acid were added. The tubes were stirred for 30 min at 250 rpm using a horizontal shaking device (IKA KS260 basic Stirrer, Staufen, Germany). Ten mL of acetonitrile were added into of tube containing soaked sample and vigorously stirred for 30 min at 250 rpm. In the next step, 4 g MgSO_4_ and 1 g NaCl were added and shaken for 30 s in vortex and then centrifuged for 10 min at 5 °C and 5000 rpm using Eppendorf Centrifuge 5810R (Eppendorf, Hamburg, Germany). Then, 2 mL of acetonitrile extract were added to 0.1 g of C18 silica sorbent and 0.3 g of MgSO_4_ for purification before centrifugation (5000 rpm) for 10 min. The purified extract was filtered through a syringe nylon filter and transferred into a vial for the LC-MS/MS analysis and for GC-MS/MS analysis the supernatant was collected and evaporated to dryness under a gentle nitrogen flow.

### 5.4. GC–MS/MS Analysis

Before GC-MS/MS analysis, 50 µL of BSA + TMCS + TMSI (3:2:3) was added to the dry extract and left 30 min at room temperature. Then, 200 µL of hexane was added, mixed thoroughly on vortex for 30 s, and washed with 1 ml of phosphate buffer (60 mM, pH 7) and mixed until the upper layer was clear. Finally, the hexane layer was transferred to an autosampler vial.

Gas chromatographic determination was carried out using a GC system Agilent 7890A coupled with an Agilent 7000A triple quadruple mass spectrometer with inter electron-impact ion source (EI, 70 Ev) and Agilent 7693 auto sampler (Agilent Technologies, Palo Alto, CA, USA). Quantitation data were acquired at selection reaction monitoring (SRM). The transfer line and source temperatures were 280° and 230°, respectively. The collision gas for MS/MS experiments was nitrogen, and the helium was used as quenching gas, both at 99.999% purity supplied by Carburos Metálicos S.L. (Barcelona, Spain). Analytes were separated on a HP-5MS 30 m × 0.25 mm × 0.25 µm capillary column. One microliter of the final clean extract of mycotoxins was injected in splitless mode in program able temperature vaporization (PTV) inlet at 250 °C, employing helium as the carried gas at fixed pressure of 20.3 psi. The oven temperature started at 80 °C and increased to 245 °C at 60 °C/min, held there for 3 min, and then increased to 260 °C progressively at 3 °C/min and finally to 270 °C at 10 °C/min and then held for 10 min. Data were acquired and processed using Agilent Masshunter version B.04.00 software (Agilent Technologies, Palo Alto, CA, USA, 2015).

### 5.5. LC–MS/MS Analysis

LC-MS/MS analyses were conducted on a system consisting of an Agilent 1200 chromatographic system (Agilent Technologies, Palo Alto, CA, USA) coupled to a 3200 QTRAP^®^ mass spectrometer (AB SCIEX, Foster City, CA, USA) equipped with a turbo electrospray ionization (ESI) interface. Separation of analyte was performed using a Gemini-NX LC-column (Phenomenex, Aschaffenburg, Germany) (150 mm × 4.6 mm, 5 µm of particle size) preceded by a guard column. The flow rate was set to 0.8 mL min^−1^, and the oven temperature was 40 °C. The two elution mobile phases were made up of the water slightly acidified with 5 mM ammonium formate and 0.1% formic acid (mobile phase A) and methanol acidified with 5 mM ammonium formate 0.1% formic acid (mobile phase B). The elution gradient started with 0% of eluent B, increased to 100% in 10 min, decreased to 80% in 5 min and, finally, decreased to 100% in 10 min, decreased to 80% in 5 min and, finally, decreased to 70% in 2 min. The column was, readjusted to the initial conditions and equilibrated for 7 min. The volume of the injections was 20 µL.

The analysis was performed using the Turbo Ion Spray instrument in positive ionization mode (ESI+). Nitrogen served as the nebulizer and collision gas. The operating conditions for the analysis were the following: ion spray voltage, 5500 V; curtain gas, 20 (arbitrary units); GS1 and GS2, 50 and 50 psi, respectively and probe temperature (TEM), 450 °C.

### 5.6. Method Validation

The method was validated for linearity, accuracy, repeatability (intraday and interday), following the EU Commission Decision 2002/657/EC [21], with food mixture of cereals, fish, legume, vegetable, and meat samples. In order to determine the linearity, calibration curves for each studied mycotoxin were constructed from standards and from extract of blank samples of cereals, fish, legume, vegetables, and meat previously analyzed and did not contain any studied compound. Linear range was tested at eight concentration levels from 0.15 to 200 µg/kg. Matrix-matched calibration curves were built by spiking blank samples with select mycotoxins after the extraction process. Both external calibration curves and matrix-matched calibration curves were constructed by plotting peak areas against concentration and linear functions were applied to the calibration curves. Matrix effect (ME) was assessed for each analyte by comparing the slope of the standard calibration curve (slope_with standard_) with that of the matrix-matched calibration curve (slope_with matrix_), for the same concentration levels.

Sensitivity was evaluated by limit of detection (LOD) and limit of quantification (LOQ), which were estimated for a signal-to-noise ratio (S/N) ≥3 and ≥10, respectively, from chromatograms of samples spiked at the lowest level validated. LODs were estimated using an extract of a blank of the different matrix (previous analyzed and negative for the mycotoxin included in this study), fortified with decreasing concentrations of the analytes, where the response of the qualifier ion was at least 3 times the response of the blank extract (*n* = 9). The LOQs were estimated in the same way as the LODs, but using criterion of S/N ≥ 10 for the qualifier ion.

Accuracy was evaluated through recovery studies and was determined calculating the ratio of the peak areas for each mycotoxin by analyzing the samples spiked before and after extraction at three addition levels 25, 50, and 100 µg/kg for all mycotoxins analyzed (AFB_1_, AFB_2_, AFG_1_, AFG_2_, OTA, FB_1_, FB_2_, ENA, ENA_1_, ENB, ENB_1_, BEA, STG, ZON, α-ZAL, β-ZAL, α-ZOL, β-ZOL, DON, 3-ADON, 15-ADON, DAS, NIV, FUS-X, NEO, T-2, and HT-2 toxins). Intra-day precision and inter-day precision (repeatability) were also investigated.

The precision of the method was determined by repeatability (*n* = 3) and reproducibility (*n* = 9) studies, and expressed as the relative standard deviation (RSD, %). The intra-day precision was expressed as the standard deviation of the recovery values of the spiked samples measured during the same day (*n* = 3). The inter-day precision was determined by analyzing the spiked samples for three different days (*n* = 9).

## Figures and Tables

**Table 1:** Summary of maximum levels of some mycotoxin in foodstuffs.

Mycotoxin	Food Product	Maximum Levels (µg/kg)
AFB_1_	Processed cereal-based foods and baby foods for infants and young children, dietetic food destined for physical treatment, cereals and cereal products, dried fruit, groundnuts and nuts, and spices	0.1–8.0
Sum of AFs	Cereal and cereal-based products, dried fruit, coffee, groundnuts, and nuts	4.0–15.0
OTA	Processed cereal-based foods and baby foods for infants and young children, dietetic food destined for physical treatment, cereals and cereal products, dried vine fruit, wine, coffee, grape juice	0.5–10
DON	Processed cereal-based products and baby foods for infants and young children, cereals intended for direct human consumption, cereal flour, bran and germ as end product marketed for direct human consumption, pasta dry, bread, pastries, biscuits, cereal snacks, and breakfast cereals	200–750
ZEA	Processed maize-based products and baby foods for infants and young children, maize and maize-based products, bread, and other unprocessed cereal	20–200
Sum of FBs	Processed maize-based products and baby foods for infants and young children, maize-based products, and unprocessed maize	200–2000

AFB_1_; Aflatoxin B_1_; AFs: Aflatoxins; OTA: ochratoxin A; DON: deoxynivalenol; ZEA: zearalenone; FBs: fumonisins.

**Table 2:** Analytical performance of the proposed method: mass spectrometry transitions, LOD, LOQ, ME, and recovery range for the different food matrices studied.

Mycotoxin	Transitions		Cereals		Legumes		Fish		Vegetables		Meats		ME	Recovery
	Quantitative	Qualitative	LOD	LOQ	LOD	LOQ	LOD	LOQ	LOD	LOQ	LOD	LOQ	(%)	(%)
	Q	Q	µg/kg	µg/kg	µg/kg	µg/kg	µg/kg	µg/kg	µg/kg	µg/kg	µg/kg	µg/kg		
OTA	404 > 102 ^a^	404 > 239	1.5	5	1.5	5	1.5	5	1.5	5	1.5	5	73–121	71–91
AFB_1_	313 > 241 ^a^	313 > 289	0.15	0.5	0.15	0.5	0.15	0.5	0.15	0.5	0.3	1	65–106	67–98
AFB2	315 > 286 ^a^	315 > 259	0.3	1	0.3	1	0.3	1	0.3	1	0.3	1	63–77	67–116
AFG_1_	329 > 243 ^a^	329 > 311	0.3	1	1.5	5	0.3	1	0.3	1	0.15	0.5	58–117	79–102
AFG_2_	331 > 313 ^a^	331 > 245	0.15	0.5	0.3	1	0.3	1	0.3	1	0.3	1	58–97	67–90
FB_1_	722 > 334 ^a^	722 > 352	1.5	5	1.5	5	1.5	5	0.3	1	1.5	5	61–141	69–114
FB_2_	706 > 336 ^a^	706 > 318	1.5	5	1.5	5	1.5	5	0.3	1	1.5	5	59–102	65–116
ENA	699 > 228 ^a^	699 > 210	0.15	0.5	0.3	0.5	0.3	1	0.15	0.5	0.15	0.5	65–117	74–114
ENA_1_	685 > 214 ^a^	685 > 210	0.15	0.5	0.15	0.5	0.15	0.5	0.15	0.5	0.15	0.5	91–125	62–104
ENB	657 > 196 ^a^	657 > 214	0.15	0.5	0.3	1	0.15	0.5	0.15	0.5	0.3	1	68–101	69–111
ENB_1_	671 > 214 ^a^	671 > 228	0.15	0.5	0.15	0.5	0.15	0.5	0.15	0.5	0.15	0.5	74–96	69–119
BEA	801 > 784 ^a^	801 > 244	0.15	0.5	0.15	0.5	0.15	0.5	0.15	0.5	0.3	1	58–103	78–99
STG	325 > 281 ^a^	325 > 310	1.5	5	1.5	5	0.3	1	1.5	5	1.5	5	70–116	71–102
DON	392 > 259 ^b^	407 > 197	0.15	0.5	0.15	0.5	0.15	0.5	0.15	0.5	0.15	0.5	82–114	75–107
3-ADON	392 > 287 ^b^	467 > 147	0.75	2.5	0.15	0.5	0.15	0.5	1.5	5	0.15	0.5	78–173	89–117
15-ADON	392 > 217 ^b^	392 > 184	0.75	2.5	0.15	0.5	0.15	0.5	0.15	0.5	0.15	0.5	77–148	73–98
NIV	289 > 73 ^b^	379 > 73	1.5	5	0.75	2.5	1.5	5	1.5	5	0.75	2.5	82–153	60–79
NEO	252 > 195 ^b^	252 > 167	0.15	0.5	0.15	0.5	0.75	2.5	0.15	0.5	0.15	0.5	77–175	91–114
DAS	350 > 229 ^b^	378 > 124	0.75	2.5	0.75	2.5	0.75	2.5	0.75	2.5	0.15	0.5	60–144	60–102
FUS-X	450 > 260 ^b^	450 > 245	0.75	2.5	0.75	2.5	0.75	2.5	1.5	5	0.15	0.5	78–151	68–106
T-2	350 > 244 ^b^	350 > 229	0.75	2.5	0.75	2.5	0.75	2.5	0.75	2.5	0.75	2.5	96–151	73–107
HT-2	347 > 157 ^b^	347 > 185	0.15	0.5	0.15	0.5	0.15	0.5	0.75	2.5	0.15	0.5	73–137	73–113
ZEA	462 > 151 ^b^	462 > 333	0.15	0.5	0.15	0.5	1.5	5	0.15	0.5	1.5	5	65–158	67–104
α-ZAL	433 > 309 ^b^	433 > 295	0.15	0.5	0.15	0.5	0.75	2.5	0.15	0.5	0.15	0.5	73–155	66–117
β-ZAL	307 > 292 ^b^	307 > 277	0.75	2.5	1.5	5	1.5	5	1.5	5	1.5	5	62–132	70–89
α-ZOL	305 > 289 ^b^	305 > 73	1.5	5	1.5	5	1.5	5	1.5	5	0.75	2.5	58–145	63–77
β-ZOL	536 > 446 ^b^	536 > 333	0.15	0.5	1.5	5	1.5	5	0.75	2.5	1.5	5	71–128	63–79

^a^ LC-MS/MS determination. ^b^ GC-MS/MS determination.

**Table 3:** Detected concentrations of DON, ENB, HT-2, and AFG_2_ in ready-to-eat food samples.

Samples	Concentration µg/kg RSD (%)			
	DON	ENB	HT-2	AFG_2_
**Cereals**				
Lasagne	2.84 ± 2.2	12.25 ± 3.2	34.43 ± 0.6	n.d.
Macaroni	5.27 ± 4.2	9.83 ± 1.0	n.d.	n.d.
Spaghetti pesto	21.59 ± 1.0	11.51 ± 6.2	7.23 ± 4.5	n.d.
Noodles	4.75 ± 10.5	86.32 ± 12.2	18.85 ± 1.6	n.d.
Rice (Paella)	n.d.	n.d.	n.d.	2.84
Rice salad	2.61 ± 0.2	n.d.	n.d.	n.d.
Pizza	3.39 ± 4.8	n.d.	9.06	n.d.
Quiche	4.1 ± 1.0	14.68 ± 4.2	n.d.	n.d.
**Vegetable**				
Vegetable soup	n.d.	n.d.	n.d.	n.d.
Garlic soup	6.19 ± 0.2	n.d.	n.d.	n.d.
Cream of leek	n.d.	n.d.	n.d.	n.d.
Gratin broccoli	n.d.	n.d.	n.d.	n.d.
Scrambled spinach	n.d.	n.d.	n.d.	n.d.
Sauteed artichoke	n.d.	n.d.	n.d.	n.d.
**Fish**				
Grilled salmon	n.d.	n.d.	n.d.	n.d.
Grilled tuna	n.d.	n.d.	n.d.	n.d.
Baked perch fillets	n.d.	n.d.	n.d.	n.d.
Hake in white wine	n.d.	n.d.	n.d.	n.d.
Grilled sole	n.d.	n.d.	n.d.	n.d.
**Legume**				
Lentils	n.d.	n.d.	n.d.	n.d.
Broad beans	6.98 ± 0.2	n.d.	n.d.	n.d.
Red beans	n.d.	n.d.	n.d.	n.d.
Cream of chickpeas	n.d.	n.d.	n.d.	n.d.
**Meats**				
Grilled chicken breast	n.d.	n.d.	n.d.	n.d.
Grilled pork loin	n.d.	n.d.	n.d.	n.d.

n.d.: not detected.

**Table 4:** Occurrence, mean, and range (minimum and maximum) of mycotoxins detected in ready-to-eat food samples.

Mycotoxin	Parameter	Cereals	Vegetables	Legumes	Total
		(*n =* 8)	(*n =* 6)	(*n =* 4)	(*n =* 25)
DON	Occurrence (%)	7 (88)	1 (17)	1 (25)	9 (36)
	Mean µg/kg	6.36	6.19	6.98	6.51
	Range (min-max) µg/kg	2.61–21.59	6.19	6.98	2.61–21.59
ENB	Occurrence (%)	5 (63)	-	-	5 (20)
	Mean µg/kg	26.91	n.d.	n.d.	26.91
	Range (min-max) µg/kg	9.83–86.32	n.d.	n.d.	9.83–86.32
HT-2	Occurrence (%)	4 (50)	-	-	4 (16)
	Mean µg/kg	17.39	n.d.	n.d.	17.39
	Range (min-max) µg/kg	9.06–34.43	n.d.	n.d.	9.06–34.43
AFG_2_	Occurrence (%)	1 (13)	-	-	1 (4)
	Mean µg/kg	2.84	n.d.	n.d.	2.84
	Range (min-max) µg/kg	2.84	n.d.	n.d.	2.84

n.d.: not detected.

**Table 5:** Mycotoxin reduction by cooking methods.

Mycotoxin	Food	Reduction (%)	Reference
DON	Pasta	67–80	[13]
DON	Pasta	25–75	[37]
DON	Pasta	13–58	[38]
DON	Pasta	40–50	[39]
ENB	Pasta	17–19	[39]
ENB	Pasta	14–65	[40]
AFs	Rice	51–95	[41]
AFs	Rice	24.8	[42]
AFB1	Rice	78–88	[43]
